# S100 Proteins As an Important Regulator of Macrophage Inflammation

**DOI:** 10.3389/fimmu.2017.01908

**Published:** 2018-01-05

**Authors:** Chang Xia, Zachary Braunstein, Amelia C. Toomey, Jixin Zhong, Xiaoquan Rao

**Affiliations:** ^1^College of Health Science and Nursing, Wuhan Polytechnic University, Wuhan, China; ^2^Cardiovascular Research Institute, Case Western Reserve University, Cleveland, OH, United States; ^3^Boonshoft School of Medicine, Wright State University, Dayton, OH, United States; ^4^Department of Health Sciences, University of Missouri, Columbia, MO, United States

**Keywords:** S100 proteins, inflammation, tissue repair, biomarkers, inflammatory disease, macrophages

## Abstract

The S100 proteins, a family of calcium-binding cytosolic proteins, have a broad range of intracellular and extracellular functions through regulating calcium balance, cell apoptosis, migration, proliferation, differentiation, energy metabolism, and inflammation. The intracellular functions of S100 proteins involve interaction with intracellular receptors, membrane protein recruitment/transportation, transcriptional regulation and integrating with enzymes or nucleic acids, and DNA repair. The S100 proteins could also be released from the cytoplasm, induced by tissue/cell damage and cellular stress. The extracellular S100 proteins, serving as a danger signal, are crucial in regulating immune homeostasis, post-traumatic injury, and inflammation. Extracellular S100 proteins are also considered biomarkers for some specific diseases. In this review, we will discuss the multi-functional roles of S100 proteins, especially their potential roles associated with cell migration, differentiation, tissue repair, and inflammation.

## Introduction

The S100 proteins, belonging to a calcium-binding cytosolic protein family, are composed of 25 known members (1–4). They have a broad range of intracellular and extracellular functions encompassing regulation cell apoptosis, proliferation, differentiation, migration, energy metabolism, calcium balance, protein phosphorylation, and inflammation (5–8).

Based on their functional roles, s100 proteins are categorized into three main subgroups: S100 proteins that only exert intracellular functions, S100 proteins that have both intracellular and extracellular roles, and S100 proteins that mainly possess extracellular effects (7). The S100 proteins within the first subgroup only exert intracellular functions. For example, S100A1 is predominantly expressed in striated muscle (especially cardiac muscle) (9) and only exert intracellular regulatory effects such as regulating SR Ca^2+^ recycle and enhancing the gain of the calcium-induced calcium release (CICR) cascade (10–12). In addition to intracellular roles, some S100 proteins are released into the extracellular environment and may exert extracellular functions. S100B in this subgroup was known to directly interact with nuclear Dbf2-related protein kinase (NDR kinase) and block the recruitment of its substrates to NDR kinase (13). Furthermore, extracellular S100B could also activate extracellular signal-regulated protein kinase (ERK) and NFκB in chondrocytes by binding to its cell surface receptor, receptor for advanced glycation end products (RAGE) (14). The third subgroup of S100 proteins such as S100A15 mainly exerts extracellular regulatory functions. These of S100 proteins are considered as potential therapeutic targets for various human disorders, including arthritis, cancer, and Alzheimer’s disease (15, 16).

**Figure 1:**
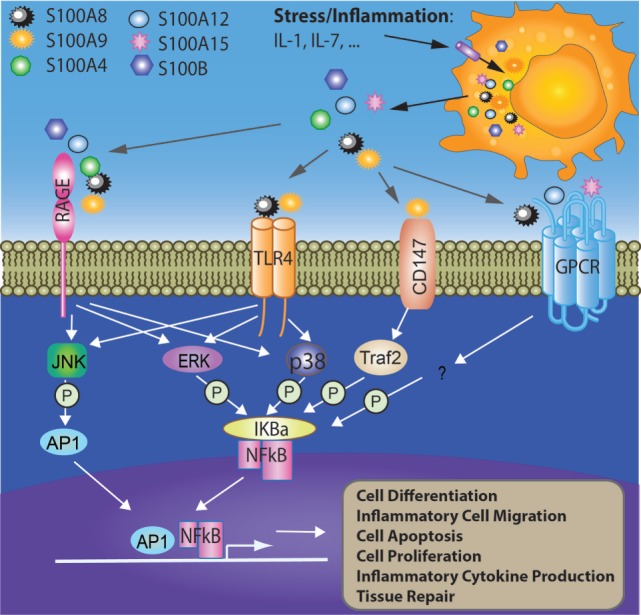
Involvement of S100 proteins in stress and inflammation-mediated responses. Cell stress or inflammation induce the release of S100 proteins to acellular compartment where they bind cell surface receptors such as RAGE, TLR4, CD147, and GPCR. The interactions between S100 proteins and their receptors activate intracellular signaling pathways such as AP1 and NFκB, which further initiates multiple cellular processes such as cell differentiation, migration, apoptosis, proliferation, and inflammation. AP1, activator protein 1; ERK, extracellular signal-regulated protein kinase; GPCR, G-protein-coupled receptor; IL-1, interleukin 1; IL-7, interleukin 7; IκBα, nuclear factor of kappa light polypeptide gene enhancer in B-cells inhibitor alpha; JNK, c-Jun N-terminal kinase; P38, p38 mitogen-activated protein kinase; RAGE, receptor for advanced glycation end products; TLR4, toll-like receptor 4; Traf2, TNF receptor-associated factor 2.

S100 proteins are involved in multiple intracellular functions which include: interacting with intracellular receptors or molecule subunits (17), membrane protein recruitment and transportation, transcriptional regulation (18, 19), regulating enzymes, nucleic acids, and DNA repair (20, 21) (Figure 1). There are two critical steps for S100 protein activation: Ca^2+^ binding (22) and homo- or hetero-dimer formation (23). Each S100 protein forming the dimer participates in ion (Ca^2+^, Zn^2+^, or Cu^2+^) binding. Ca^2+^ also contributes to the formation of S100 protein oligomers, especially calprotectin (S100A8/A9 tetramer) (22, 24, 25).

When released to the extracellular space, S100 proteins have crucial activities in the regulation of immune homeostasis, post-traumatic injury, and inflammation. S100 proteins trigger inflammation through interacting with receptors RAGE and TLR4 (26). Increasing evidence has demonstrated that calprotectin (S100A8/A9) is an endogenous agonist of TLR4 (26). Binding to TLR4 initiates a signaling cascade and regulates inflammation, cell proliferation, differentiation, and tumor development in an NF-κB-dependent manner (8, 26–28). Apart from TLR4, RAGE has also been suggested to bind S100 proteins such as S100A7, S100A12, S100A8/A9, and S100B (27, 29–31). By interacting with RAGE, S100 proteins activate NF-κB, inducing the production of pro-inflammatory cytokines leading to the migration of neutrophils, monocytes, and macrophages (30, 31). In addition to the NF-κB pathway, MAP kinase-mediated signaling is also induced by S100 proteins such as S100P (32, 33). Interestingly, S100A6 activates RAGE and promotes apoptosis, while S100B inactivates RAGE by interacting with the basic fibroblast growth factor and its receptor (14, 34). Extracellular S100 proteins may regulate the apoptosis, proliferation, differentiation, and migration of a number of cell types including monocytes, macrophages, neutrophils, lymphocytes, myoblast, epithelial cells, endothelial cells, smooth muscle cells, neurons, and fibroblasts. In this review, we aim to summarize the immune regulatory role of S100 proteins and their potential involvement in inflammatory regulation, tissue repair, and tumorigenesis.

## S100 Genes and Molecular Structure

Each S100 family protein is encoded by a separate gene. Most S100 genes are located within the chromosome 1q21 with a few exceptions. For example, S100A11P is located within chromosome 7q22-q3, S100B in located within chromosome 21q22, S100P is located in chromosome 4p16, S100G is located in chromosome Xp22, and S100Z is located with chromosome 5q13 (5). The sequence homology among S100 proteins varies from 22 to 57%, which is mainly due to the variance at the hinge region and C-terminus, the regions associated with their function (35).

S100 proteins are small proteins with a molecular weight of 10–12 kDa. Each S100 protein consists of two EF-hand helix–loop–helix structural motifs, which are arranged in a back-to-back manner and linked with a flexible hinge (23). The activity of the proteins is regulated by metal ions (such as calcium, zinc, and copper), which modulates the folding and oligomerization of the protein (36, 37).

## Expression Pattern and Regulation

Epigenetic mechanisms play a key role in the regulation of S100 protein expression. S100A3, S100A10, S10011, and S100P could be detected in various medulloblastoma cell lines treated with DNA de-methylation (38). It is reported that DNA hypomethylation could induce S100A6 overexpression in gastric cancer. Lower levels of CpG methylation in the first intron and second exon regions of the S100A6 gene, accompanied by higher levels of acetylated histone H3 binding to the promoter, have been reported in the gastric cancer tissues (39). Lower methylation in the proximal promoter region of the S100P gene was also found in prostate cancer cell lines (40). The expression of S100 proteins may also be regulated by micro RNAs, although further studies are needed to provide direct evidence. NFAT5, a transcription factor that initiates S100A4 expression (41), is regulated by miR-568 (42).

The expression of S100 proteins is strictly regulated to maintain immune homeostasis (7, 43). S100A8 and S100A9 are predominately expressed in monocytes, neutrophils, and dendritic cells (44, 45). However, they are also expressed in various other types of cells upon activation, such as fibroblasts (46), mature macrophages (47), vascular endothelial cells (48–50), and keratinocytes (51). In neutrophils, 45% of the cytosolic proteins are constituted with S100A8 and S100A9, whereas the proportion is only 1% in monocytes (52). The expression levels in different monocyte subsets also vary. The level of S100A8 mRNA is higher in classical CD14^+^/CD16^−^ human monocytes when compared to non-classical CD14^+^CD16^+^ monocytes (47).

Increasing evidence indicates that the expression of most S100 proteins is different between physiological and pathological conditions. The expression of S100A8 and S100A9 could be upregulated by a number of conditions such as oxidative stress, specific cytokines, and growth factors in many types of cells (53). S100A12 is mainly expressed in neutrophils, monocytes, and early macrophages (53, 54), but it can also be detected in endothelial cells, keratinocytes, epithelial cells, and pro-inflammatory macrophages under inflammatory conditions (51, 55–58). In human epidermal keratinocytes, interleukin (IL)-1α induces a significant increase of S100A9 expression by the p38 MAPK pathway (59). The expression of S100A5 is upregulated in bladder cancers (60). Pro-inflammatory cytokines could increase S100A7 expression in human breast cancer (61). IL-17, IL-22, and bacterial products (e.g., flagellin) can enhance S100A7 expression in keratinocytes (62). IL-6 and IL-8 released from myofibroblasts could also trigger the upregulation of S100A8/A9 in tumor-infiltrated myeloid cells (63). S100A9 was significantly higher in the peripheral blood in patients with implant-associated osteomyelitis. S100A9 expressing cells were also increased in tissue biopsies from patients with implant infections, compared with the non-infected individuals (64).

## S100 Proteins Function as Damage-Associated Molecular Pattern (DAMP) Molecules

In addition to serving as calcium-binding proteins, S100 proteins were later discovered as DAMP molecules (26, 65, 66). DAMPs were considered as a series of intracellular molecules linked with cell death and tissue damage through inducing a rapid inflammatory response or production biologically active molecules (67, 68). DAMPs are biomolecules that are released from damaged or stressed cells and could act as endogenous danger signal to activate inflammatory response (69). S100 proteins could be released from the cells after cell damage/stress or activation of phagocytes such as neutrophils and macrophages. The extracellular S100 proteins then become danger signals and activate immune cells and endothelial cells by binding to the pattern recognition receptors such as TLRs and RAGE.

They play an important role in modulating inflammatory responses (70). Once released from the cell, calprotectins function as an endogenous agonist to bind TLR4 (S100A8/A9 and S100A12) (26) and RAGE (S100A8/A9 and S100A7) [(6, 31, 71) #3535]. In the site of inflammation, calprotectin acts as a chemotactic factor by inducing neutrophils adhesion (72). Furthermore, S100A8/A9 induces apoptosis and autophagy in various cell types such as lymphocytes, macrophages, endothelial cells, and tumor cells (73). It has been shown that reactive oxygen species (ROS) is the critical factor in S100A8/A9-induced cell death and involves BNIP3. The increase of ROS production in mitochondria subsequently causes mitochondrial damage and lysosomal activation (73).

## S100 Proteins in Macrophages Migration, Invasion, and Differentiation

It is widely accepted that macrophages contribute to immune defense, immune regulation, and tissue repair. Based on their cytokine production and activation conditions, macrophages are categorized into two populations: pro-inflammatory M1 (classically activated macrophage) and anti-inflammatory M2 (alternatively activated macrophage). Calprotectin could induce pro-inflammatory cytokine production in monocytes and macrophages through NF-κB and p38 MAPK pathways (74). An increasing number of findings demonstrate that S100 proteins contribute to the adhesion and migration of leukocytes. For example, the release of S100A8/A9 has been suggested to facilitate monocyte and neutrophil transmigration (75, 76). The S100A8/A9 heterodimer enhances the expression of β2 integrin CD11b and the ability of adhesion in phagocytes (72, 77). Moreover, the response of S100A9^−/−^ monocytes to chemotaxis was reduced when compared with wild-type cells. For example, IL 8-induced CD11b upregulation was abolished in S100A9^−/−^ monocytes and neutrophils (78). S100A4 has also been shown to interact with cytoskeletal proteins to promote cell migration and deletion of *s100a4*, which leads to the deficiency of macrophage migration and chemotactic reactions (79–81). S100A12 induced the production of pro-inflammatory cytokine IL-6 and IL-8 in both a dose-dependent and time-dependent manner. This was critical to regulate the recruitment of monocytes and TNF-α release (82).

The intimate relationship between macrophages and cancer cells plays a crucial role in tumor growth and metastasis. Tumor associated macrophages influence tumor growth by modulating local inflammation, inhibiting antitumor immunity, and stimulating angiogenesis (83–85). It is commonly accepted that macrophages contribute to tumor growth and invasion. They are recruited to the site of tumors *via* chemoattractants such as CCL3-8, vascular endothelial growth factor (VEGF), and macrophage inflammatory protein-1 alpha (MIP-1α) (86). The monocytes or macrophages tend to differentiate into the M2 macrophage phenotype rather than the tumoricidal M1 phenotype, producing pro-tumor cytokines, such as macrophage colony-stimulating factor, IL-10, IL-4, and IL-13 (83, 87, 88). S100A10 was shown to mediate the migration of macrophages to the tumor site. Tumor growth was reduced in S100A10-null mice, compared with wild-type mice, and was accompanied by less macrophages within the tumor. There were many macrophages throughout the tumor in wild-type mice, where macrophages were observed only around the absolute tumor tissue border in S100A10-null mice (89). Intraperitoneal injection of wild-type macrophages restored macrophage density within the tumor, but injection of S100A10-deficient macrophages did not. Interestingly, intratumoral injection of macrophages of either genotype could rescue tumor growth, suggesting that S100A10^−/−^ macrophages still have the ability to stimulate tumor growth but lack the ability to invade into the tumor (89). Another study showed that S100A10 deficiency decreased plasmin generation and matrix metalloproteinase 9 activation in macrophages, both of which are associated with macrophage invasion and migration (90).

Downregulation of S100A8 and S100A9 is associated with the differentiation of myeloid cells toward dendritic cells and macrophages (91, 92). S100A8 and S100A9 are co-expressed in fetal myeloid progenitors, with its expression level associated with the development of the myeloid lineage (93). They are highly expressed in monocytes and neutrophils. However, the expressions of S100A8 and S100A9 are lost when monocytes terminally differentiate into tissue macrophages (93). Recent data have shown that S100A8 can be induced by oxidative stress in macrophages in an IL-10-dependent manner (51). Interestingly, S100A8/A9 has also been shown to control the cell cycle (94). S100A9 inhibited myeloid cells differentiation through generation of ROS (92). S100A9 is able to induce the differentiation of monocytes toward the osteoclast type in *in vitro* culture experiments and S100A9 derived from neutrophils and S100A9-induced osteoclast generation were considered as important reasons for bone degradation in infectious osteomyelitis (95). S100A8 and S100A9 have also been shown to mediate the arresting effect of TNF-α on the differentiation of immature myeloid-derived suppressor cells into dendritic cells and macrophages in a RAGE-dependent manner (96). Consistent with this finding, IL-6 and IL-8 released from myofibroblasts in tumor microenvironment upregulate S100A8/S100A9 in myeloid cells and induce the differentiation of myeloid cells into S100A8/S100A9-expressing myeloid-derived suppressor cells and M2 macrophages (63).

## Role of S100 Proteins in Tissue Repair

Damage-associated molecular pattern molecules play a critical role in tissue repair. S100A7, S100A8/A9, S100A12, and S100A15, well-documented DAMPs, have been shown to participate inflammatory tissue damage and tissue repair. The link between S100A12 and the severity of coronary and carotid atherosclerosis has been evidenced by multiple human studies (97–99). S100A7 is highly expressed in the skin, and the expression is increased in inflamed skin, which has been shown to be induced by pro-inflammatory cytokines (IL-17 and IL-22) and bacterial products such as flagellin (62), that the increase of S100A7 has been associated with multiple inflammatory skin diseases, such as psoriasis and atopic dermatitis (62, 100). Similarly, the expression of S100A15 was amplified in the epidermis of psoriatic lesions and acted as chemoattractants for immune cells (101). S100A8/A9 exerts anti-inflammatory function in healthy state, while oxidative stress-associated pathological conditions activate their pro-inflammatory functions (102). Increased plasma S100A8/A9 levels have been associated with atherogenesis, plaque vulnerability, myocardial infarction (MI), cardiovascular death, and heart failure. In a mouse model of angiotensin-induced cardiac damage, it was shown that S100A8/A9 released by granulocytes upregulated pro-inflammatory gene expression and induced the release of cytokines and chemokines in a RAGE-dependent manner. This process promoted myocardial tissue inflammation and fibrotic scar formation (103, 104). In a mouse model of collagenase-induced arthritis, the expression of S100A8 and S100A9 in synovial was upregulated in wild-type mice. In addition, *S100a9*^−/−^ mice were protected from collagenase-induced synovitis, cartilage degradation, and osteophyte formation (105, 106). S100A9 antibodies could block the accumulation of fibroblasts and decrease fibrosis in local inflammatory microenvironment (104). In contrast, S100A1 or S100A4, released following MI, has a beneficial effect following heart injury by promoting muscle tissue repair and maintaining contractility (107, 108).

Binding of S100B to RAGE and the subsequent increase of angiogenic factor VEGF have been shown to be essential in the development of macular degeneration (109). In addition, S100B activates the Ras-MEK-ERK1/2-NF-κB pathway in neural cells and leads to the activation of small GTPases, Rac1/Cdc 42, and neurite growth (110). In vascular smooth muscle cells, S100B induces the upregulation of ROS and recruits JAK2 and STAT3, which results in the proliferation of vascular smooth muscle cells (111). Similarly, S100B also increased cellular proliferation though activating the Phosphatidylinositol-4,5-bisphosphate 3-kinase-AKT pathway in a RAGE-dependent manner (14). On the other hand, S100B could induce apoptosis by increasing production of ROS and the release of cytochrome-c from mitochondria (110). High levels of S100B are released from injured cardiomyocytes following MI and could promote cell apoptosis through RAGE. Also, S100B released from injured skeletal muscle tissue could stimulate myoblast proliferation but inhibit myoblast differentiation by activating bFGF/FGFR1 signaling (112, 113). However, the regeneration effects of S100B on the injured myoblasts are strongly dependent on cell density, because it triggers RAGE, but not bFGF/FGFR1 signaling, at an early stage of low-density myoblast differentiation (114).

## The Role of S100 Proteins in Inflammatory Diseases

S100 proteins, particularly calgranulins, play a significant role in mediating innate and acquired immune responses, which contribute to the development of chronic inflammatory diseases.

Calgranulins are associated with joint inflammation in patients with rheumatoid arthritis (RA) (115). The level of S100A8/A9 in the serum and synovial fluid was significantly increased in RA (116, 117). Recent findings showed that S100A8/A9 was upregulated in early but not late phase osteoarthritis (OA) (118). S100A8/A9 plasma levels were increased at baseline in human OA participants. Meanwhile, osteophyte size was drastically reduced in S100A9^−/−^ mice-induced OA (106). It has also been confirmed that S100A8/A9 contributes to cartilage degradation and development of inflammatory arthritis in an antigen-induced arthritis model (119). Similar to S100A8/A9, human S100A7 and S100A15 were first confirmed as over-expressed in psoriatic plaques (120). Increasing evidence supports an association of S100A7 with several inflammatory skin diseases, including psoriasis and atopic dermatitis (62, 100). Evidence strongly indicates that S100A8/A9 levels are higher in hypercalprotectinemia, an extremely rare inflammatory disorder (121–123). Although the mechanism is still unclear, it is possible that the releasing of extracellular S100A8/A9 is dysregulated, which accounts for the abnormal increase of calprotectin and subsequent hyperactive inflammatory reaction. It is suggested that S100 proteins are involved in interacting with both the immune system and the pathogen. S100A12 plays a key role in fighting infections. For example, it has been shown that S100A12 plays a critical role in anti-parasite responses (124). In addition to directly killing *Mycobacterium tuberculosis* and *Mycobacterium leprae*, S10012 is also required for TLR2/1L- and IFN-γ-induced antimicrobial activity against *Mycobacterium* (125). Haley et al. also showed that S100A12 can help to repress the biogenesis and activity of *H. pylori* cag type IV secretion system by binding nutrient zinc, which results in suppressed bacterial growth and viability (126).

S100A8, S100A9, and S100A12 are abundantly expressed by neutrophils. Evidence indicates that these three members of S100 proteins are released by neutrophils, inducing MUC5AC production in airway epithelial cells through activating TLR4 and RAGE signaling pathway. This reveals the relationship between chronic neutrophilic inflammation and obstructive airway diseases such as severe asthma, COPD, and cystic fibrosis (127). In correlation with their role in the development of chronic inflammation, S100A8/A9 also participates in the hyperglycemia-induced increase of myelopoiesis occurring in a RAGE-dependent manner in diabetic mice (128). Interestingly, the amount of circulating monocytes and neutrophils were decreased when antidiabetic treatment normalized the glycemic index of Ldlr^−/−^ atherosclerotic mice, which might explain the increased severity of atherosclerosis found in patients with diabetes (128). In accordance with these findings, increased serum concentrations of S100A8/A9 were detected in obese individuals (129). Furthermore, the expression of the macrophage marker CD68 was increased in the visceral adipose tissue (130). Some research of dipeptidyl peptidase-4 inhibitors for the treatment of type 2 diabetes mellitus indicates that vildagliptin could increase the mRNA expression levels of S100A9 and TNF-α in human hepatocytes. In addition, it may induce the release of S100A8/A9 complex from HL-60 cells *via* TNF-α-independent manner, which might be a contributing factor of vildagliptin-associated liver dysfunction (131).

## S100 Proteins as Biomarkers in Specific Diseases

Extracellular S100 proteins are involved in the activation of G protein-coupled receptors, heparan sulfate proteoglycans or N-Glycans, and scavenger receptors in autocrine and paracrine manners (132, 133). Since S100A proteins can be detected in body fluids, such as urine, cerebrospinal fluid, serum, sputum, and feces, extracellular S100 proteins are considered as biomarkers associated with certain diseases (134–137).

It has been suggested that S100A12, S100A8/A9, and S100B are linked to specific diseases and conditions such as auto-inflammatory diseases, stroke, and trauma (138). The level of S100A12 in the blood is increased in the patients with diabetes, which is correlated with a higher risk of cardiovascular disease development (139). Bogdanova et al. detected the serum concentration of S100A12 and other acute-phase inflammatory markers in thirty-five patients with periodic disease (PD) (140). The level of S100A12 in PD was significantly higher compared to other familial periodic fevers. S100A12 was more sensitive to assess the subclinical activity of autoinflammatory diseases, when compared to other inflammatory biomarkers such as neutrophil counts, fibrinogen, C-reactive protein (CRP), and erythrocyte sedimentation rate (140). Similarly, the serum concentrations of S100A12, as a novel biomarker, were shown to be upregulated in patients with Familial Mediterranean fever in comparison to controls (141).

The plasma concentrations of S100A9 were significantly higher in patients with implant-associated infectious osteomyelitis when compared to patients with sterile inflammation or healthy individuals. In addition, S100A9 was associated with osteoclast generation and bone degradation. Therefore, it could serve as a novel diagnostic marker to aid in the differential diagnosis (95). Similarly, serum levels of S100A8 and S100A9 were dramatically increased in IL-1Ra^−/−^ mice and contributed to bone erosion, cartilage damage, and synovial inflammation. Thus, they can be considered as a systemic or local biomarker to evaluate the extent of inflammation and inflammatory joint destruction in seronegative arthritis (142). It was shown that the expression of S100A8/A9 was high in human atherosclerotic lesions and the blood levels were also increased in the patients with coronary artery diseases (CAD), which implied S100A8/A9 might act as a biomarker for cardiovascular events (143). Recent research has shown similar findings that serum S100A8/A9 levels were elevated in 178 CAD patients with unstable angina pectoris or acute myocardial infarction, and the level of S100A8/A9 was significantly positively linked with CRP (*P* < 0.01) (144). These clinical data suggest that S100A8/A9 may become a novel biomarker for CAD (139).

In addition, more studies explored the value S100A8/A9 as a predictive biomarker for autoimmune diseases. In RA, S100A8/A9 was suggested as a potential biomarker in predicting clinical response to monitor treatment (145, 146). Some clinical investigations have indicated that S100A8/A9 levels might be a more sensitive predictor for monitoring synovial inflammation in RA patients when compared with other markers such as CRP levels (147).

The study by Shakeri et al. suggested that S100 B protein could be used as a posttraumatic biomarker for predicting brain death in severely injured patients with exclusive head trauma during the first 6 h after trauma, but found no relationship between S100 B levels and death (148). Pelinka et al. confirmed that *in vitro* S100 B concentrations increased significantly in rats with femoral fractures but not head injury (149). Interestingly, adverse results indicated that there was no difference in S100 B concentrations between patients with and without head injury (150). S100B has also been considered as a prognostic marker of the acute phase of neurologic damage (151), predicting the outcome of traumatic brain injury and large volume cerebral infarction (152, 153). The level of serum S100B in ischemic stroke implied a worse outcome secondary to the stroke (154, 155). This research demonstrates that S100 B is correlated to trauma and a worse long-term outcome. S100B has recently been confirmed to be associated with some genetic disorders and was found to be over-expressed in patients with Down syndrome (156, 157). There was also study showing that S100B may be one of the best biomarkers of melanoma (158).

## S100 Proteins as Therapeutic Targets in Disease

Although direct clinical evidence is limited, increasing studies indicate that S100 proteins may also serve as a therapeutic target for certain disease conditions. As mentioned above, S100 proteins are involved in a number of diseases including inflammatory disease. It has been reported that multiple anti-allergic drugs such as amlexanox, cromolyn, and tranilast are able to bind S100A12 and S100A13, and block downstream RAGE signaling (159). Therefore, these drugs may serve as a therapeutic approach to target S100 proteins. Multiple S100 proteins such as S100A4 (160) and S100B (161) have been shown to participate in the neoplastic disorders by binding to P53 and suppressing its phosphorylation (162). Therefore, efforts are being made to restore P53 function by targeting S100 proteins (163). In an *in vitro* study, Most et al. demonstrated that extracellular S100A1 is endocytosed by the neonatal ventricular cardiomyocytes protects cardiomyocytes from 2-deoxyglucose and oxidative stress-induced apoptosis *via* activation of ERK (164). Adeno-associated virus-mediated S100A1 gene transfer in failing cardiomyocytes was also shown to be able to restore the contractile function, suggesting a potential implication of AAV-mediated S100A1 gene therapy in heart failure (165, 166). Despite the promising potentials, the feasibility and safety of these approaches and issues such as how to control and keep expression levels in the therapeutic window need to be further investigated (166).

## Conclusion

Evidence strongly supports that S100 proteins, as a remarkable multifunctional proteins family, are involved in the regulation of several important biological processes such as the inflammatory response, protecting the intra- and extracellular environments during infection, cell proliferation and differentiation, tumor growth and metastasis, cell apoptosis, energy, and glutathione metabolism.

However, the activities of all members S100 proteins depend on the cell-specific expression patterns and binding targets even the local microenvironment. Extracellular effects of S100 proteins interact with receptors including TLR-4, RAGE, and heparan sulfate proteoglycans during infection and inflammation which associated with the pathogenesis of inflammatory such as autoimmune disease, infectious diseases, allergy, tumorigenesis and metastasis, and anti-microbial disease. Extracellular S100 proteins can also contribute to the regulation of tissue development and regeneration or repair, which is essential for elucidating their role in the pathological procession of tissue damage, cell apoptosis, or tissue repair.

Although growing evidence has begun to show the regulation of S100 proteins in detail which improves our understanding of how immune homeostasis is maintained during the development of S100 protein-associated disease, there are certain gaps in our understanding of the role of S100 proteins in pathophysiology. Among 25 known members of S100 family, only limited number of S100 proteins such as S100A8 and S100A9 have been well documented and the functional roles of other members are underappreciated. In addition, further studies are required to fully reveal the underlying mechanisms by which S100 proteins participate in a variety of disease conditions. For instance, a role of S100P has been reported in leukemia (167), while the exact function of S100P in leukemia and the signal pathways involved in this process are not completely understood. Also, the direct clinical evidence of the therapeutic potential of S100 proteins is limited at current stage. Therefore, future directions in this area could focus on the development of therapeutic approaches targeting S100 proteins, verification of the therapeutic potential of S100 proteins in both preclinical and clinical settings, and elucidation of the underlying mechanisms.

## Author Contributions

CX and JZ reviewed the literature and wrote the first draft. ZB, AT, JZ, and XR reviewed the literature and finalized the manuscript.

## Conflict of Interest Statement

The authors declare that the research was conducted in the absence of any commercial or financial relationships that could be construed as a potential conflict of interest.
